# A propensity score-adjusted analysis of efficacy of high-flow nasal oxygen during awake tracheal intubation

**DOI:** 10.1038/s41598-022-15608-6

**Published:** 2022-07-04

**Authors:** Hye Jin Kim, Min-Soo Kim, So Yeon Kim, In Kyung Min, Wyun Kon Park, Sei Han Song, Dongkwan Shin, Hyun Joo Kim

**Affiliations:** 1 Department of Anesthesiology and Pain Medicine, Anesthesia and Pain Research Institute, Yonsei University College of Medicine, 50-1 Yonsei-ro, Seodaemun-gu, Seoul, 03722 Republic of Korea; 2 Biostatistics Collaboration Unit, Department of Biomedical Systems Informatics, Yonsei University College of Medicine, 50-1 Yonsei-ro, Seodaemun-gu, Seoul, 03722 Republic of Korea

**Keywords:** Health care, Medical research

## Abstract

Oxygen supplementation is crucial for awake tracheal intubation (ATI) using a flexible bronchoscope in patients with an anticipated difficult airway. However, the modality of optimal oxygen delivery remains unclear. This retrospective study compared high-flow nasal oxygen (HFNO) and conventional low-flow oxygen supply during ATI. We applied inverse probability of treatment weighting (IPTW) to account for biases due to clinical characteristic differences between the groups. The primary endpoint was the lowest oxygen saturation during ATI. The secondary endpoints were incidence of desaturation, multiple attempts, failure rate, and procedural duration. After IPTW adjustment, the lowest oxygen saturation in the HFNO group during ATI was significantly higher than that in the conventional oxygenation group (99.3 ± 0.2 vs. 97.5 ± 0.5, *P* < 0.001). Moreover, the HFNO group had fewer cases with multiple attempts than the conventional oxygenation group (3% vs. 16%, *P* = 0.007). There were no significant differences between the two groups in the incidence of desaturation, failure and procedural duration. Our findings suggest that HFNO was associated with improved lowest oxygen saturation and a lower rate of multiple attempts during ATI. Therefore, we recommend using HFNO for safer oxygen delivery and improved quality of procedure during ATI.

## Introduction

Transnasal humidified rapid-insufficient ventilatory exchange (THRIVE) allows apneic oxygenation with warmed and humidified oxygen through a nasal cannula. It decreases the nasopharyngeal dead space and creates a low positive pressure due to the high-flow oxygen-based flushing effect, which facilitates oxygenation^1,2^. The technique showed positive outcomes in conscious and sedated patients in the intensive care unit, emergency room, procedure room, and those under general anesthesia for surgery^3–7^. Recently, its utility in the prevention and treatment of hypoxia has expanded its scope of use.

Anesthetic induction suppresses spontaneous respiration and decreases the laryngopharyngeal muscle tone in the oropharynx. This might exacerbate intubation difficulty and eventually increase the risk of hypoxia in patients whose airways are difficult to manage^4^. Therefore, awake tracheal intubation (ATI) is the gold standard for such patients^8^. The Difficult Airway Society recommended supplemental oxygen during ATI at the Grade B level^9^. During ATI with a flexible bronchoscope, the incidence of hypoxia (defined as oxygen saturation < 90%) was reported to be 20.9% when low-flow oxygen (2–4 L/min) was administered through a nasal cannula^10^. However, Badiger et al. reported a hypoxia incidence of 0% in patients receiving high-flow nasal oxygen (HFNO) during ATI^4^, suggesting that HFNO might prevent the occurrence of hypoxia. In contrast, a prospective, observational cohort study reported no difference in the incidence of hypoxia between patients receiving HFNO or other oxygen administration strategies^11^. However, this study focused on describing the performance of the entire ATI procedure; therefore, equal distribution of baseline characteristics between cases with and without HFNO was not obtained. As the application of HFNO during ATI is gradually increasing worldwide^11^, further research is required to determine whether HFNO is superior to the conventional oxygenation technique.

This retrospective study compared HFNO with conventional oxygenation techniques (low-flow oxygen supply through a standard nasal cannula or a simple face mask) in patients undergoing ATI. The inverse probability of treatment weighting (IPTW) was conducted using propensity scores to account for differences in baseline characteristics between the groups. The primary endpoint was the lowest oxygen saturation during ATI.

## Methods

### Study design and ethical approval

This single-center, retrospective cohort study was approved by the Institutional Review Board of Severance Hospital, Seoul, South Korea (Approval Number: 4-2021-0901; August 21, 2021). The requirement for written informed consent was waived due to the retrospective nature of the study. This study followed the guidelines of the Declaration of Helsinki (1964) and its later amendments (2013).

### Patients

We included patients who underwent ATI for surgery at Severance Hospital, Yonsei University Health System, Seoul, Korea, between March 2017 and May 2021. We excluded the following patients: those who underwent other procedures, for example, arterial line cannulation, before ATI and after entering the operation room, and those with incomplete data on oxygen saturation, attempt numbers, and time taken for ATI. However, we did not exclude cases where the time taken for ATI could not be specified due to failed ATI.

The patients were divided into HFNO and conventional oxygenation groups. The conventional oxygenation group received low-flow oxygen through a standard nasal cannula or a simple face mask.

### Data collection

We retrospectively reviewed the electronic medical records of the included patients and retrieved the following baseline characteristics: age, sex, height, weight, body mass index, nature of the operation (emergency or elective), American Society of Anesthesiologists physical status classification, history of hypertension, coronary arterial occlusive disease, congestive heart failure, chronic obstructive pulmonary disease, and previous radiotherapy and/or surgery for head and neck cancer.

We also recorded pre-procedural data, including airway bleeding, need for oxygen supplementation before entering the operating room, and initial oxygen saturation at the time of entering the operating room.

Moreover, we recorded intra-procedural data on oxygen saturation, intravenously administered drugs for ATI (glycopyrrolate, remifentanil, dexmedetomidine, or propofol), intra-procedural position of the patient (supine or sitting), HFNO utilization, performance of trans-tracheal block, intubation performer, intubation route (nasal or oral), multiple attempts (more than two attempts), duration of ATI (interval between entry to the operating room and ATI completion), successful or failed ATI, and occurrence of adverse effects (barotrauma or direct contact airway injury) during ATI.

### Study endpoints

The primary endpoint was the lowest oxygen saturation during ATI. The secondary endpoints were incidences of desaturation (oxygen saturation < 90%), multiple attempts, failure, and the procedural duration.

### Statistical analysis

Continuous variables were analyzed using an independent t-test. Categorical variables were analyzed using the chi-square test or Fisher’s exact test. Numerical data were presented as mean (standard deviation). Categorical variables were presented as counts and percentages. Statistical analysis was performed using IBM SPSS Statistics for Windows, Version 26.0 (IBM Corp., Armonk, NY, USA), R version 3.6.0 (The R Foundation for Statistical Computing, Vienna, Austria), and SAS version 9.4 (SAS Inc., Cary, NC, USA). Statistical significance was set at *P* < 0.05.

The IPTW method was used to balance the groups for the baseline characteristics of the patients and the procedural environments. We estimated the propensity scores using a multiple logistic regression model based on the following variables: age, sex, body mass index, nature of the operation (emergency or elective), presence of chronic obstructive pulmonary disease, pre-procedural airway bleeding, need for oxygen supplementation before entering the operating room, drugs used for ATI (glycopyrrolate, remifentanil, dexmedetomidine, or propofol), intra-procedural patient position (supine or sitting), performance of trans-tracheal block, intubation performer, and intubation route (nasal or oral). The between-group balance was assessed using the absolute standardized differences (ASDs). Variables with an ASD < 0.2 were considered to have an acceptable balance^12^. We used stabilized weights to reduce variability in the IPTW analysis.

After the IPTW adjustment, continuous variables were analyzed using an adjusted independent t-test by weighting the individual contributions, while categorical variables were analyzed using the Pearson chi-squared test after the Rao–Scott second-order correction. Continuous outcome variables were further analyzed using a multivariable linear regression model by weighting the individual contributions to correct for the confounding effect of variables with ASD > 0.1. The regression model approach was not applied for the categorical outcome variables due to the small number of events.

## Results

**Figure 1:**
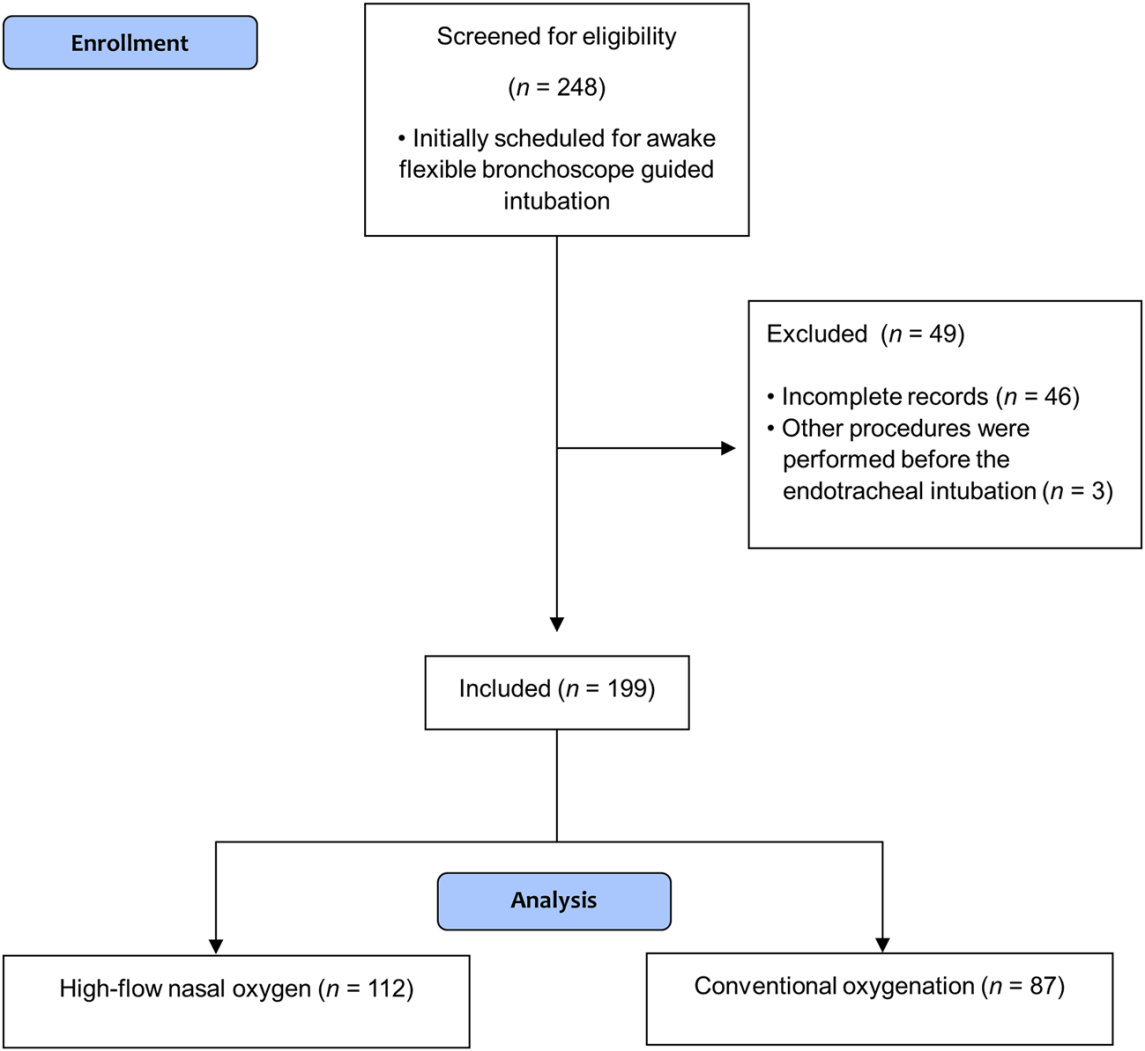
Flow chart of patient selection. Of the 248 patients who underwent awake tracheal intubation (ATI) between March 2017 and May 2021 at our institution, we excluded 46 due to incomplete data on oxygen saturation, the number of attempts, and ATI duration, and three who underwent other procedures before the ATI. Finally, 199 patients were included.

We screened 248 patients who underwent ATI between March 2017 and May 2021, and excluded 49 patients based on the exclusion criteria. Finally, 199 patients were included in the analysis (Fig. 1). Of these, data for the duration of the ATI were missing for five patients because the ATI procedure had failed. These five patients were analyzed for all outcomes except the time taken for ATI. Distribution of surgical procedures in the HFNO and conventional oxygenation groups is shown in Supplementary Table S1.

Before IPTW adjustment, patients in the HFNO group were more likely to undergo an emergency operation and were more frequently diagnosed with hypertension than those in the conventional oxygenation group. However, the baseline characteristics were similar for both the groups after IPTW adjustment (Table 1).

**Table 1:** Baseline patient characteristics before and after IPTW adjustment.

Characteristic	Before IPTW			After IPTW			
	HFNO group (*n* = 112)	Conventional oxygenation group (*n* = 87)	*P*-value	HFNO group (*n* = 157)	Conventional oxygenation group (*n* = 124)	*P*-value	ASD
**Patient characteristic**							
Age (years)	59.6 (14.7)	57.8 (13.7)	0.376	58.7 (1.7)	56.7 (2.7)	0.531	0.131
Male sex	75 (67%)	61(70%)	0.636	115 (83%)	89 (72%)	0.864	0.031
BMI (kg/m^2^)	22.1 (3.9)	22.2 (3.6)	0.876	21.6 (0.5)	22.2 (0.5)	0.342	0.187
Emergency	28 (25%)	11 (13%)	0.029	25 (16%)	12 (10%)	0.184	0.191
**Underlying medical data**							
ASA-PS classification (1/2/3/4)	5/25/78/4 (4/22/70/4%)	4/18/63/2 (5/21/72/2%)	0.966	7/28/118/3 (5/18/75/2%)	4/27/91/2 (3/22/74/2%)	0.828	0.136
Hypertension	48 (43%)	21 (24%)	0.006	60 (39%)	36 (29%)	0.352	0.197
CAOD	6 (5%)	4 (5%)	> 0.999	5 (3%)	10 (8%)	0.254	0.191
CHF	0 (0%)	0 (0%)	N/A	0 (0%)	0 (0%)	N/A	
COPD	10 (9%)	7 (8%)	0.825	19 (12%)	14 (11%)	0.903	0.030
Previous radiotherapy for head and neck cancer	38 (34%)	28 (32%)	0.795	52 (33%)	31 (25%)	0.365	0.176
Previous surgery for head and neck cancer	56 (50%)	44 (51%)	0.936	78 (50%)	62 (50%)	0.969	0.008
**Pre-procedural data**							
Airway bleeding (none/little/severe)	109/0/3 (97/0/3%)	83/1/3 (95/1/3%)	0.648	151/0/6 (96/0/4%)	119/1/4 (96/1/3%)	0.682	0.109
Oxygen supplementation^a^	14 (13%)	6 (7%)	0.192	15 (10%)	7 (6%)	0.333	0.144
Initial oxygen saturation (%)	98.5 (1.5)	98.8 (1.6)	0.311	98.7 (0.2)	98.9 (0.2)	0.408	0.136

Values are presented as means (standard deviations) or counts (percentages).

*IPTW* inverse probability treatment weighting, *HFNO* high-flow nasal oxygen, *ASD* absolute standardized difference, *BMI* body mass index, *ASA-PS* American Society of Anesthesiologists-physical status, *CAOD* coronary artery occlusive disease, *CHF* congestive heart failure, *N/A* not applicable, *COPD* chronic obstructive pulmonary disease.

^a^Defined as the need for oxygen supplementation before entering the operating room.

Table 2 shows the intra-procedural data during ATI. Before IPTW adjustment, the HFNO group had more patients who were administered with glycopyrrolate, remifentanil, and propofol, while the conventional oxygenation group had more patients who were administered with dexmedetomidine. Additionally, a trans-tracheal block was performed less frequently, and the oral approach was the more preferred intubation route in the HFNO group than in the conventional oxygenation group. After IPTW adjustment, the intra-procedural data were similar for both the groups.

**Table 2:** Intra-procedural data before and after IPTW adjustment.

Intra-procedural data	Before IPTW			After IPTW			
	HFNO group (*n* = 112)	Conventional oxygenation group (*n* = 87)	*P*-value	HFNO group (*n* = 157)	Conventional oxygenation group (*n* = 124)	*P*-value	ASD
**Drug administered**							
Glycopyrrolate	95 (85%)	53 (61%)	< 0.001	117 (75%)	85 (69%)	0.589	0.123
Remifentanil	99 (88%)	21 (31%)	< 0.001	96 (61%)	70 (57%)	0.678	0.094
Dexmedetomidine	4 (4%)	22 (25%)	< 0.001	16 (10%)	18 (15%)	0.538	0.150
Propofol	28 (25%)	2 (2%)	< 0.001	23 (15%)	12 (10%)	0.608	0.147
Sitting position	7 (6%)	3 (3%)	0.518	9 (6%)	6 (5%)	0.858	0.035
Trans-tracheal block	10 (9%)	53 (61%)	< 0.001	42 (27%)	44 (36%)	0.419	0.195
Intubation performer (resident/fellow/staff/airway specialist staff^a^)	4/10/8/90 (4/9/7/80%)	4/13/9/61 (5/15/10/70%)	0.403	5/22/19/110 (3/14/12/70%)	5/16/14/89 (4/13/11/72%)	0.991	0.054
Intubation route (nasal/oral)	39/73 (35/65%)	48/39 (55/45%)	0.004	58/98 (37/63%)	57/66 (46/54%)	0.376	0.185

Values are presented as counts (percentage).

*IPTW* inverse probability treatment weighting, *HFNO* high-flow nasal oxygen, *ASD* absolute standardized difference.

^a^Airway specialist staff refers to a staff professor who performed > 50 awake intubations.

After IPTW adjustment, the mean lowest oxygen saturation in the HFNO group [99.3% (0.2%)] was higher than that in the conventional oxygenation group [97.5% (0.5%); Table 3]. Moreover, the HFNO group had a lower proportion of cases with multiple attempts than that in the conventional oxygenation group (3% vs. 16%, *P* = 0.007). There were no significant between-group differences in the time taken for ATI, occurrence of desaturation, and failed ATI.

**Table 3:** Analysis of outcomes after IPTW adjustment.

Outcome	HFNO group	Conventional oxygenation group	*P*-value
Lowest oxygen saturation (%)	99.3 (0.2)	97.5 (0.5)	< 0.001
Change in oxygen saturation from the initial to the lowest oxygen saturation^a^	0.6 (0.3)	− 1.4 (0.5)	< 0.001
Desaturation	0 (0%)	4 (3%)	0.078
Multiple attempts	5 (3%)	20 (16%)	0.007
Failed intubation	2 (1%)	3 (2%)	0.409
Procedural duration^b^	23.5 (1.4)	26.9 (1.5)	0.095

Values are presented as means (standard deviations) or counts (percentage).

*IPTW* inverse probability treatment weighting, *HFNO* high-flow nasal oxygen, *ATI* awake tracheal intubation.

^a^Calculated as [lowest oxygen saturation during ATI with oxygen supplementation] − [oxygen saturation at the time of entry into the operating room].

^b^Time from entry into the operating room to the completion of awake intubation.

Correction of the confounding effects of variables with ASD > 0.1 revealed a significant between-group difference in the lowest oxygen saturation (IPTW-adjusted mean difference: 1.742%; standard error: 0.520, *P* = 0.001; Table 4). Similar to the previous results (Table 3), no significant between-group difference in the IPTW-adjusted time taken for ATIs was noted between the two groups (IPTW-adjusted mean difference: − 3.026 min; standard error: 0.890; *P* = 0.111).

**Table 4:** Analysis of continuous outcome variables after IPTW and correction of variables with ASD > 0.1^a^.

Outcome	Group	Mean % difference (standard error)	*P*-value
Lowest oxygen saturation	Conventional oxygenation	Reference	0.001
	HFNO	1.742 (0.520)	
Procedural duration^b^	Conventional oxygenation	Reference	0.111
	HFNO	−3.026 (0.890)	

*IPTW* inverse probability treatment weighting, *ASD* absolute standardized difference, *HFNO* high-flow nasal oxygen.

^a^Variables with ASD > 0.1 after IPTW included age, body mass index, emergency operation, pre-procedural airway bleeding, need for oxygen supplementation before entering the operating room, drugs used for awake tracheal intubation (glycopyrrolate, dexmedetomidine, propofol), trans-tracheal block, and intubation route.

^b^Time from entry into the operating room to completion of awake tracheal intubation.

## Discussion

In this retrospective study, the use of HFNO during ATI was associated with improved level of intra-procedural lowest oxygen saturation and fewer multiple attempts. However, the intubation time, the incidences of desaturation and intubation failure were similar, regardless of the use of HFNO.

Our findings demonstrated that compared to conventional oxygen supply with a facial mask or nasal cannula, HFNO improved the lowest oxygen saturation during ATI. Moreover, oxygen saturation decreased after the start of ATI in 40% of the patients in the conventional oxygenation group. Conversely, only 10% patients experienced decreased oxygen saturation during ATI when HFNO was used. Our finding is consistent with a previous observational study, which reported that immediate post-procedural oxygen saturation was maintained or improved in 50 patients who underwent ATI using HFNO^4^. Similarly, HFNO with spontaneous ventilation reportedly enhances oxygenation under moderate or deep sedation for procedures other than ATI^3,5^. A randomized controlled study of 30 patients who underwent dental treatment by Sago et al. reported that the lowest oxygen saturation in patients who received oxygen at 5 L/min through a nasal cannula was significantly lower than those who received oxygen at 30 or 50 L/min using THRIVE^3^. Douglas et al. investigated 60 patients who underwent bronchoscopy and reported that oxygen delivery at 30–70 L/min using THRIVE was associated with an improved lowest oxygen saturation than the standard oxygen therapy of 10 L/min through a bite block (97.5% [94–99%] vs. 92% [88–95%]; *P* < 0.001)^5^. Therefore, HFNO can feasibly improve oxygen saturation compared to conventional oxygen supply during ATI.

Numerous studies have reported that HFNO increases pharyngeal oxygen concentration^13–17^. Additionally, the continuous positive airway pressure generation effect may improve oxygenation with HFNO. A study on healthy volunteers found that when a gas flow of 60 L/min was administered through HFNO with the participants’ mouth open and closed, the expiratory pharyngeal pressures were 2.7 cm H_2_O and 7.4 cm H_2_O, respectively^18^. Moreover, warm and humid air improves mucociliary clearance and reduces patient discomfort^19^. We believe that these properties of HFNO contribute to improved oxygen saturation during ATI.

We observed no difference between the groups with regards to the occurrence of desaturation. This could be attributed to the low incidence of desaturation (2%) in our study. Moreover, only 11% of the patients required oxygen supply before entering the operating room, and the initial oxygen saturation was relatively stable [98.6% (range, 93–100%)], which could have contributed to the lack of a beneficial effect of HFNO on desaturation. Similarly, Douglas et al. reported that HFNO did not prevent the occurrence of desaturation in patients undergoing bronchoscopy, which was defined as oxygen saturation < 90%^5^. Nonetheless, none of the patients who received HFNO in our study experienced desaturation; however, it occurred in 3% of the patients in the conventional oxygenation group. Therefore, even if there was no statistically significant difference in the incidence of desaturation between the two groups, the advantage of HFNO in stable maintenance or improvement of oxygen saturation during ATI should not be overlooked.

The use of HFNO in our study was associated with a reduction in the rate of multiple attempts. This finding is similar to the result reported by Sago et al., wherein HFNO reduced the intra-procedural interruptions during dental treatment under sedation^3^. HFNO probably makes procedures less disruptive and easier to succeed because it stabilizes the patients’ physiologic status by improving the oxygenation, such that they can better adapt to the procedure. Moreover, fewer multiple attempts at intubation might help prevent airway injury^20^. The airway passage in patients requiring ATI is often narrowed due to anatomical abnormalities, head and neck cancer, or inflammation. Better airway patency, achieved when using the nasal route, may lead to better oxygenation^21^, enabling patients to better tolerate the procedure and thereby reducing the number of attempts. Fewer attempts could also be related to the operator's skill and their being more calm on observing that the patients are well saturated^22,23^.

Similar to previous reports, we found an intubation failure rate of 1–2%^11,24,25^ and no significant difference between the groups. It appears that improved oxygenation could not resolve intubation failure in our study, since four of the five patients with intubation failure had the lowest oxygen saturation of 100%, while it was 94% in the fifth patient. We speculate that eventual intubation failure is determined by anatomical factors of the patient's head and neck that cause poor laryngeal visualization or difficult tube delivery.

Adverse effects such as barotrauma and direct contact airway injury were not observed in either group. A previous study reported cases of gastric rupture at an oxygen flow rate of 4 L/min via a nasal or nasopharyngeal catheter^26^, which raised concerns regarding possible serious side effects caused by HFNO. However, a prospective interventional study reported that HFNO at flow rates of up to 70 L/min for 30 min did not cause gastric distension in spontaneously breathing healthy individuals^27^. Moreover, an observational study using THRIVE reported no side effects^4^. Another prospective cohort study reported a similar incidence of complications in using HFNO or other oxygenation methods during ATI^11^. However, airway injury in patients who underwent awake craniotomy was associated with low-flow oxygen delivery (6 L/min) through the nasopharyngeal airway more often than that with HFNO at 40 or 60 L/min^28^. With careful patient selection, the incidence of adverse effects in using HFNO seems insignificant. Taken together, our findings provide evidence supporting the use of HFNO in ATI, which is consistent with the 2020 Difficult Airway Society guidelines^9^.

This study had some limitations. First, being a retrospective study, it had an inherent drawback of selection bias. The medical staff may have selected patients at a higher risk of hypoxia for HFNO application. However, we applied the IPTW method to adjust for between-group discrepancies, which minimized the selection bias. Second, because the desaturation incidence in our study was low, the statistical power might be too low to observe the effect of HFNO on preventing desaturation. Nonetheless, our findings showed the superiority of HFNO in improving the lowest oxygen saturation. Third, we did not analyze other ventilation-related parameters, including end-tidal CO_2_ and arterial partial pressure of CO_2_.

## Conclusions

Our findings suggest that HFNO during ATI helps improve oxygenation and increase the rate of successful intubation with fewer attempts. Therefore, we recommend using HFNO for safer oxygen delivery and improved quality of the procedure during ATI.

## Supplementary Information

Supplementary Information.

## Data Availability

The datasets generated for this study are available on request to the corresponding author. The data are not publicly available due to privacy reasons.
