# Supplementary material for: A propensity score-adjusted analysis of efficacy of high-flow nasal oxygen during awake tracheal intubation
Source: Sci Rep. 2022 Jul 4;12:11306. doi: 10.1038/s41598-022-15608-6 (PMC9253013; doi:10.1038/s41598-022-15608-6)
Supplement: Supplementary file 1 — Supplementary Information. [file 41598_2022_15608_MOESM1_ESM.pdf]

Supplementary data

A propensity score-adjusted analysis of efficacy of high-flow nasal oxygen during awake tracheal intubation

Hye Jin Kim [1]; Min-Soo Kim [1]; So Yeon Kim [1]; In Kyung Min [2]; Wyun Kon Park [1]; Sei Han Song [1]; Dongkwan Shin [1]; Hyun Joo Kim [1,*]

1 Department of Anesthesiology and Pain Medicine, Anesthesia and Pain Research Institute, Yonsei University College of Medicine, 50-1 Yonsei-ro, Seodaemun-gu, Seoul 03722, Korea; jackiedi@yuhs.ac (Hye J.K.); kmsviola@yuhs.ac (M.-S.K.); kimsy326@yuhs.ac (S.Y.K.); wkp7ark@yuhs.ac (W.K.P.); songseihan@yuhs.ac (S.H.S.); shindk94@yuhs.ac (D.S.)

2 Biostatistics Collaboration Unit, Department of Biomedical Systems Informatics, Yonsei University College of Medicine, 50-1 Yonsei-ro, Seodaemun-gu, Seoul 03722, Korea; iknice9@yuhs.ac (I.K.M.)

* **Correspondence**: jjollong@gmail.com; Tel.: 82-02-2224-4464

Table S1. Distribution of surgical procedures

Procedure	HFNO group (n = 112)	Conventional oxygenation group (n = 87)	P-value
Otorhinolaryngology	94 (83.9%)	65 (74.7%)	0.108
Oral-maxillofacial	12 (10.7%)	12 (13.8%)	0.508
Plastic surgery	4 (3.6%)	6 (6.9%)	0.338
Urology	1 (0.9%)	3 (3.4%)	0.320
Orthopedic	0 (0.0%)	1 (1.1%)	0.437
Gynecology	1 (0.9%)	0 (0.0%)	>0.999

Values are presented as counts (percentages).

HFNO, high-flow nasal oxygen
